# HER2-siRNA delivered by EGFR-specific single chain antibody inhibits NSCLC cell proliferation and tumor growth

**DOI:** 10.18632/oncotarget.8053

**Published:** 2016-03-14

**Authors:** Yuan Lu, Yuan Wang, Mi Zhang, Li Liu, Fakai Li, Jian Zhang, Mingxiang Ye, Hu Zhao, Jing Zhao, Bo Yan, Angang Yang, Rui Zhang, Xia Li, Xinling Ren

**Affiliations:** ^1^ Department of Respiratory Medicine, Xijing Hospital, Fourth Military Medical University, Xi’an, China; ^2^ Department of Geriatrics, Xianyang Central Hospital, Xianyang, China; ^3^ Department of Respiratory Medicine, PLA General Hospital, Beijing, China; ^4^ Organ Transplant Institute, Fuzhou General Hospital (DongFang Hospital), Xiamen University, Fuzhou, China; ^5^ State Key Laboratory of Cancer Biology, Department of Biochemistry and Molecular Biology, Fourth Military Medical University, Xi’an, China; ^6^ State Key Laboratory of Cancer Biology, Department of Immunology, Fourth Military Medical University, Xi’an, China

**Keywords:** non-small cell lung cancer, EGFR, HER2, single-chain viable-fragment antibody, small interfering RNA

## Abstract

Overexpression of human epidermal growth factor receptor type2 (HER2) is closely associated with aggressive progression and poor prognosis in non-small cell lung cancer (NSCLC). Here, we generated an EGFR-scFv-arginine nonamer peptide fusion protein (scFv-9R) as a cargo to deliver HER2 specific siRNA into HER2-positive NSCLC cells both *in vitro* and *in vivo*. HER2-siRNAs delivered by scFv-9R effeciently silenced HER2 expression in EGFR-positive NSCLC cells, and consequently resulted in G1 arrest and cell growth inhibition. Importantly, intravenous injection of scFv-9R/HER2-siRNA complex markedly suppressed growth of EGFR-positive NSCLC xenograft in nude mice, resulting from downregulated HER2 expression, reduced cell proliferation and enhanced cell apoptosis. Collectively, our study provides a novel therapeutic strategy for the treatment of EGFR-positive, HER2-overexpressed NSCLC.

## INTRODUCTION

Human epidermal growth factor receptor 2 (HER2), also known as neu or c-ErbB-2, is a member of epidermal growth factor receptor (EGFR) family and featured by no known natural ligand. HER2 can be activated by interaction with other ligand-stimulated family members, and thus plays an important role in cell growth, survival and differentiation [1]. HER2 gene amplification occurs in 3-22% of non-small cell lung cancer (NSCLC) and its expression is much higher in approximately 5-20% of NSCLC compared with normal tissues. Among all histotypes of NSCLC, lung adenocarcinoma has the highest frequency of HER2 positivity with 17-42% of total cases [2-4]. Importantly, HER2 gene amplification or overexpression in NSCLC has been established as a driver event and a poor prognostic factor [5-7]. Moreover, the synchronous expression of both EGFR and HER2 occurs in approximately 10-50 % of NSCLC patients and is also prognostic for increased recurrence risk and decreased overall survival [8-10]. These findings make HER2 as an attractive target for anti-NSCLC therapy [11]. Indeed, many HER2-targeting drugs, such as the humanized monoclonal antibody (trastuzumab and pertuzumab) [12, 13], dual inhibitor of EGFR/HER-2 (lapatinib) [14], and pan-inhibitor of EGFR/HER2/HER4 (afatinib) [15], are already undergoing clinical trials in NSCLC. However, because of limited inhibitory effects or acquired resistance, neither monoclonal antibodies nor tyrosine kinase inhibitors could be the final solution for the lung cancer treatment [16, 17].

RNA interference (RNAi) technology has been recognized as a promising strategy in cancer treatment [18]. Several early-phase clinical trials in cancer patients showed that inspirational responses have be achieved using RNAi-based approaches [19]. However, efficient delivery of siRNAs into tumor cells is still unresolved because of the characteristics as fast nuclease degradation, short circulation half-life, inadequate bio-distribution in tumor tissues and poor cellular uptake by cancer cells.

Single-chain antibody fragment (scFv) is an ideal tool for delivery of therapeutic reagents because of its similar binding affinity but less immunogenicity and stronger penetrating capability compared with the intact antibody [20]. In recent years, a rational scFv-based siRNA delivery system has been devised. It is a fusion protein composed of a scFv capable of binding to cell surface receptor, and a basic polypeptide (e.g., a nona-arginine) capable of carrying siRNAs. It has been reported that this scFv-based siRNA-deliver system can increase the therapeutic index, minimize potential side effects and reduce cellular toxicity of siRNA, representing its clinical applicability [21-25].

EGFR is overexpressed in approximately 60% NSCLC patients and HER2 aberration partially accounts for the failure of current therapeutics [26-28]. In the present study, we generated an EGFR-targeting scFv (abbreviated as an “s”) and fused it with a nine-mer arginine (9R) peptide (scFv-9R). Furthermore, we performed both *in vivo* and *in vitro* experiments to test that if this scFv-9R fusion protein can deliver siRNA into EGFR-positive NSCLC cells efficiently. Finally, we functionally analyzed alterations in cell proliferation, cell apoptosis and tumor growth upon treatment with scFv-9R/HER2-siRNA complex.

## RESULTS

### The scFv-9R fusion protein retains EGFR binding ability and internalizes into EGFR-positive NSCLC cells

A single chain antibody fragment (scFv) of EGFR fused with a nona-arginine residues (9R) and a six-histidine (6×His) tag was expressed in the recombinant vector pGEX-4T-1-ScFv-9R. This scFv is composed of amino acid sequences of nimotuzumab (a monoclonal anti-EGFR antibody) with VH and VL chains connected by a linker (Gly4Ser3). Theoretically, it can bind to EGFR on cellular surface and subsequently internalize into cells. And the part of nona-arginine residues (9R) is capable of carrying siRNA. The pGEX-4T-1-S which has only scFv fragment tagged with a six-histidine (6×His) was used as a control.

The scFv or scFv-9R fusion proteins were expressed and purified from *E. coli* cells. As shown in Supplementary Figure 1A, SDS-PAGE analysis showed that the purity of both GST-scFv and GST-scFv-9R is more than 90%. When cleaving GST fragment with thrombin, the molecular weight of fusion proteins were about 26 kDa. The recombinant proteins were further confirmed by Western blot using anti-His antibody (Supplementary Figure 1B).

To examine EGFR-binding capability of scFv and scFv-9R, we performed enzyme-linked immunosorbent assay (ELISA). The commercial recombinant proteins of EGFR were immobilized on 96-well microplates and incubated with our purified proteins. As shown in Supplementary Figure 1C, both scFv and scFv-9R showed retained antibody affinity for recombinant EGFR compared to PBS and negative control protein BSA.

**Figure 1:**
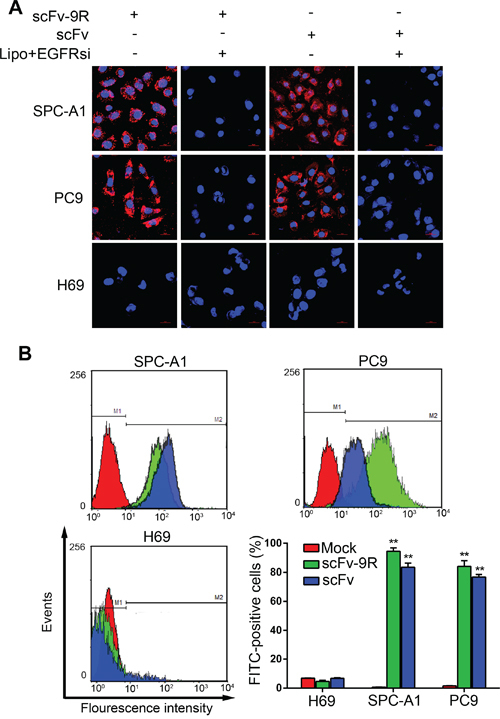
scFv-9R can internalize into EGFR-positive NSCLC cells **A.** Lung cancer cells were either transfected with EGFRsi using Lipofectamine (Lipo) or not; 48 h later, cells were incubated with scFv-9R or scFv for 6 h, and then stained with anti-His antibody (Cy3-labeled:Red) and DAPI (Blue). Distribution of scFv-9R and scFv was visualized by LSCM. Scale bars, 20 μm. **B.** Cells were incubated with scFv-9R or scFv, and then immunostained with anti-His antibody. Uptake of scFv-9R or scFv was assessed by FCM analysis. Mock represents PBS-treated control. Representative result was shown from 3 independent experiments, **p < 0.01.

Next, we examined whether scFv or scFv-9R can internalize into EGFR-positive cells. For this purpose, we added the purified scFv or scFv-9R into the culture medium of EGFR-positive SPC-A1, PC9 cells or EGFR-deficient H69 cells and then detected the distribution of the fusion proteins with immunofluorescent staining. To rule out the possibility of nonspecific effects, we also knocked down EGFR in cells before adding the fusion proteins. As shown in Figure 1A, the signal of fusion proteins was obvious on cellular membrane and cytoplasm in EGFR-positive SPC-A1 and PC9 cells, but not in EGFR-deficient H69 cells. Knockdown of EGFR in SPC-A1 and PC9 cells markedly attenuated the cellular uptake of the fusion proteins. Weaker fluorescent signal of fusion proteins in EGFR siRNA-pretransfected cells was similar to the endogenous noisy signal. Furthermore, the uptake of fusion proteins was monitored and quantified by flow cytometry (FCM) assay. The shift of FITC peak represents an increasing number of cells uptaking the fusion proteins in EGFP-positive SPC-A1 and PC9 cells but not in EGFP-negative H69 cells (Figure 1B). The positive rates of SPC-A1 and PC9 cells uptaking scFv were 83.42 ± 2.39% and 76.80 ± 1.74%, respectively, and the positive rates of these cells uptaking scFv-9R were 94.50 ± 2.37% and 84.16 ± 3.91%, respectively. However, H69 cells showed only background fluorescence, and the number of FITC-positive cells was no more than 7% in average. Collectively, these results not only confirm the EGFR-binding and internalizing capacity of the recombinant scFv and scFv-9R proteins, but also indicate that genetic fusion of scFv with 9R peptides and His tag does not alter this capacity.

### ScFv-9R efficiently and specifically delivered siRNA into EGFR-positive NSCLC cells *in vitro* and *in vivo*

To assess the siRNA-carrying ability of scFv-9R, we performed the gel retardation assay by mixing negatively charged DNA fragments with scFv-9R proteins. As shown in the Supplementary Figure 1D, scFv-9R proteins attenuated migration of DNA fragments in a dose-dependent manner, while DNA incubated with scFv or BSA showed no significant change in gel mobility, suggesting the interaction between scFv-9R and DNA is a specific conjugation and relies on the presence of 9R peptides.

**Figure 2:**
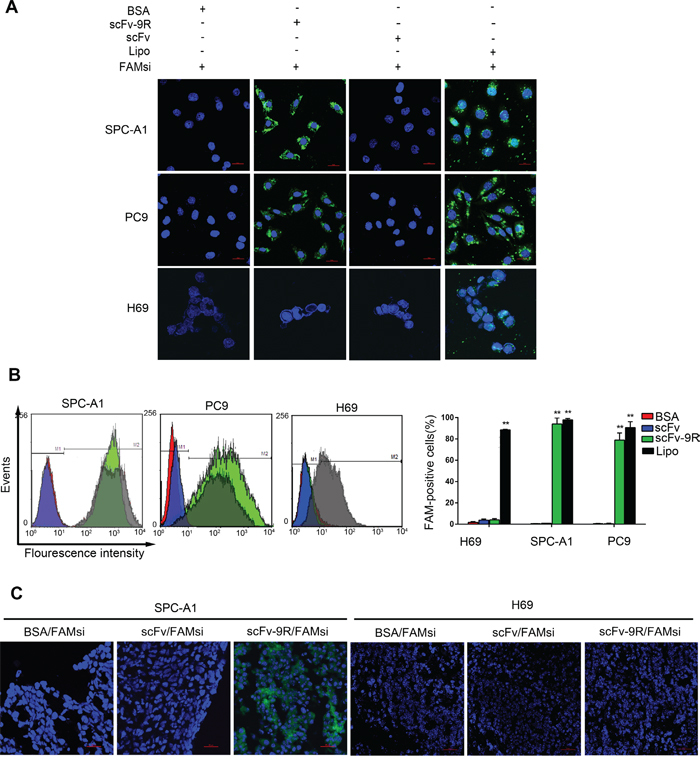
scFv-9R can specifically deliver siRNAs into EGFR-positive NSCLC cells **A** and **B.** Lung cancer cells were either incubated with pre-mixed BSA/FAMsi, scFv/FAMsi or scFv-9R/FAMsi, or transfected with FAMsi using Lipofectamine. Distribution of FAMsi (green) was visualized by LSCM (A) or quantitatively analyzed by FCM (B). **C.** Nude mice bearing SPC-A1 or H69 xenografts were intravenously treated with BSA/FAMsi, scFv/FAMsi or scFv-9R/FAMsi complex. Fluorescence images of tumor cryosections were taken by LSCM. Scale bar, 20 μm. A representative result of 3 independent experiments was shown, **p < 0.01.

Next, we incubated the cancer cells with pre-mixed scFv/FAM-labelled siRNA (FAMsi) or scFv-9R/FAMsi complex, and then observed the uptake of FAMsi. Transfected cells with FAMsi by Lipofectamine served as a positive control. As shown in Figure 2A, EGFR-positive cells incubated with the scFv-9R/FAMsi showed that the FAMsi is not only on the cellular membrane but also in the entire cytoplasm. It is almost similar to FAMsi-transfected cells with Lipofectamine. Whereas, in scFv/FAMsi and BSA/FAMsi incubated EGFR-positive cells, we did not detect obvious uptake of FAMsi. EGFR-negative H69 cells incubated with the scFv-9R/FAMsi did not show positive FAM signals. Then, we used FCM assay to test scFv-9R-mediated uptake of FAMsi in tumor cells. As shown in Figure 2B, the shift of the FAM peak occurred in scFv-9R/FAMsi-incubated SPC-A1 and PC9 cells, but not in BSA/FAMsi- or scFv/FAMsi-incubated cells, while EGFR-negative H69 cells could barely take up FAMsi pre-mixed with scFv-9R. These results indicate that scFv-9R can efficiently and specifically deliver FAMsi into EGFR-positive cells *in vitro*.

We then used SPC-A1- and H69-xengraft mouse model to evaluate efficiency and specificity of scFv-9R-mediated delivery of siRNA *in vivo*. For this purpose, FAM-labeled siRNAs (2’-O-me modified) were pre-mixed with BSA, scFv or scFv-9R and then injected into SPC-A1- or H69-bearing nude mice via the tail vein. After 48 hours, the sectional frozen slices of xenografts and normal tissues were prepared and examined using the confocal microscope. As shown in Figure 2C, FAMsi can be detected in SPC-A1 tumor slices from the mice injected with scFv-9R/FAMsi, but not in those from the mice injected with BSA/FAMsi and scFv/FAMsi. H69 tumor slices did not show any positive signal of FAMsi. Weaker fluorescence could also be detected in kidney and liver tissues, but not in other tissues like brain, heart, lung, spleen and muscle (Supplementary Figure 3). These results suggest that scFv-9R can efficiently and specifically deliver siRNAs into EGFR-positive tumors and metabolize from liver and kidney *in vivo*.

### HER2-siRNA delivered by scFv-9R effectively silenced HER2 expression in EGFR-positive NSCLC cells and inhibited cell growth *in vitro*

**Figure 3:**
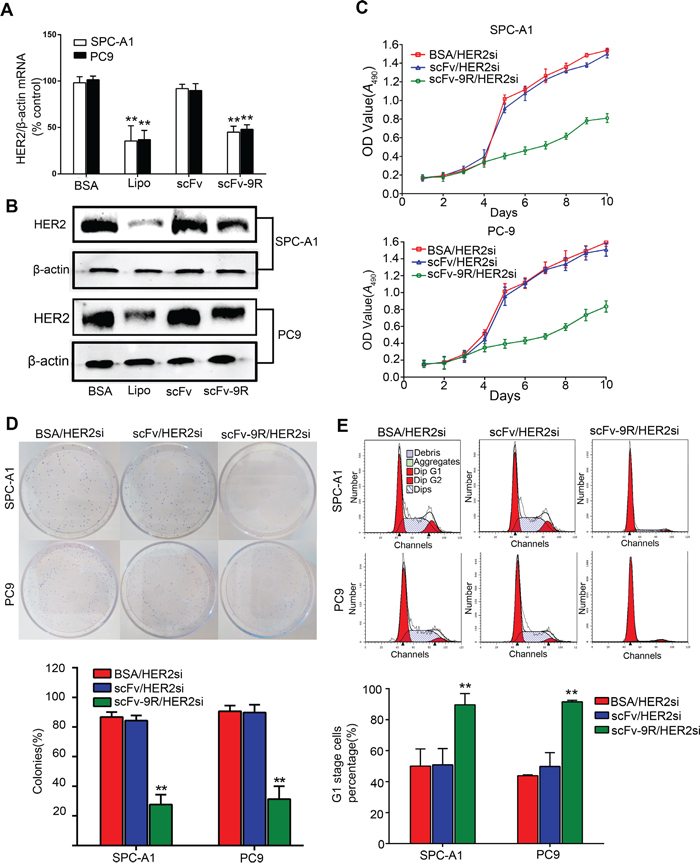
scFv-9R/HER2si suppressed HER2 expression in EGFR-positive cells and inhibited cell proliferation and colony formation **A** and **B.** Lung cancer cells were either incubated with BSA/HER2si, scFv/HER2si or scFv-9R/HER2si, or transfected with HER2si using Lipofectamine. HER2 expression was determined by qRT-PCR (A) and Western blot (B), β-action was used as internal control. **C, D** and **E.** Cells were cultured in the presence of BSA/HER2si, scFv/HER2si or scFv-9R/HER2si, and then subjected to MTT assay (C), colony-formation assay (D) and FCM analysis (E). A representative result was shown from 3 independent experiments. **p < 0.01.

Because HER2 is frequently overexpressed in EGFR-positive NSCLC and to some extent accounts for resistance to EGFR-targeted therapy [31], we attempted to utilize scFv-9R to deliver HER2 siRNA (HER2si) and examine their effect on EGFR-positive, HER2-overexpressed NSCLC. First, HER2 expression was determined by qRT-PCR and Western blot when cells were incubated with BSA/HER2si, scFv/HER2si or scFv-9R/HER2si. As shown in Figure 3A, HER2 expression levels were significantly decreased by ~45% in SPC-A1 and PC9 cells treated with scFv-9R/HER2si, which is comparable to Lipofectamine-mediated HER2si transfection (about ~60% decrease in HER2 mRNA level). The silencing effect of scFv-9R/HER2si complex on HER2 in SPC-A1 and PC9 cells were also confirmed by Western blot (Figure 3B). On the contrary, scFv/HER2si treatment did not alter HER2 expression level in these two cell lines. These results suggest that scFv-9R can specifically deliver HER2si into EGFR-positive cells and efficiently silence HER2 expression. Additionally, signaling downstream of HER2 was found to be suppressed in the two cell lines treated with scFv-9R/HER2si (Supplementary Figure 4D). Significant decrease of phosphorylated-ERK (p-ERK) and phosphorylated-Akt (p-Akt) in scFv-9R/HER2si treated cells was observed. These data suggest that the HER2 signaling pathways were interrupted by the downregulation of HER2 in SPC-A1 and PC9 cells.

Next, we examined the effect of scFv-9R/HER2si-induced HER2 silence on tumor cell growth *in vitro*. Upon treatment with BSA/HER2si, scFv/HER2si or scFv-9R/HER2si, the cell viability and clonogenic ability of SPC-A1 and PC9 were measured by MTT and colony-formation assay. As shown in Figure 3C, HER2si delivered by scFv-9R significantly inhibited cell viability but not by scFv. Furthermore, the clonogenic ratio of SPC-A1 and PC9 cells upon BSA/HER2si treatment was 86.77 ± 3.36% and 90.66 ± 3.75%, respectively, and it was 84.31 ± 3.51% and 89.74 ± 5.36% for SPC-A1 and PC9 cells treated with scFv/HER2si. But the colony formation ratio for SPC-A1 and PC9 cells treated with scFv-9R/HER2si were dramatically reduced to 27.67 ± 6.66% and 31.40 ± 8.61%, respectively (Figure 3D).

To explore the reason for the growth-inhibitory effect of scFv-9R/HER2si, we analyzed the cell cycle distribution and cell apoptosis. As shown in Figure 3E, the cell proportion in G1 phase were about 50% in both SPC-A1 and PC9 control groups treated with BSA/HER2si, however, it was increased to 89.65 ± 7.20% in SPC-A1 cells and to 91.55 ± 2.37% in PC9 cells treated with scFv-9R/HER2si. However, the cell proportion in S and G2 phase were decreased in these two cell lines treated with scFv-9R/HER2si (Supplementary Figure 4A). We also examined the effect of scFv-9R/HER2si on the cell cycle distribution in EGFR-negative H69 cells and found that scFv-9R/HER2si had no effect on it (Supplementary Figure 4B). In addition, cell apoptosis assay showed that scFv-9R/HER2si does not induce cell apoptosis in SPC-A1 or PC9 cells (Supplementary Figure 4C). These data indicate that scFv-9R/HER2si-induced G1 arrest, but not cell apoptosis, accounts for the compromised cell growth *in vitro*.

### *In vivo* antitumor activity of scFv-9R/HER2si

**Figure 4:**
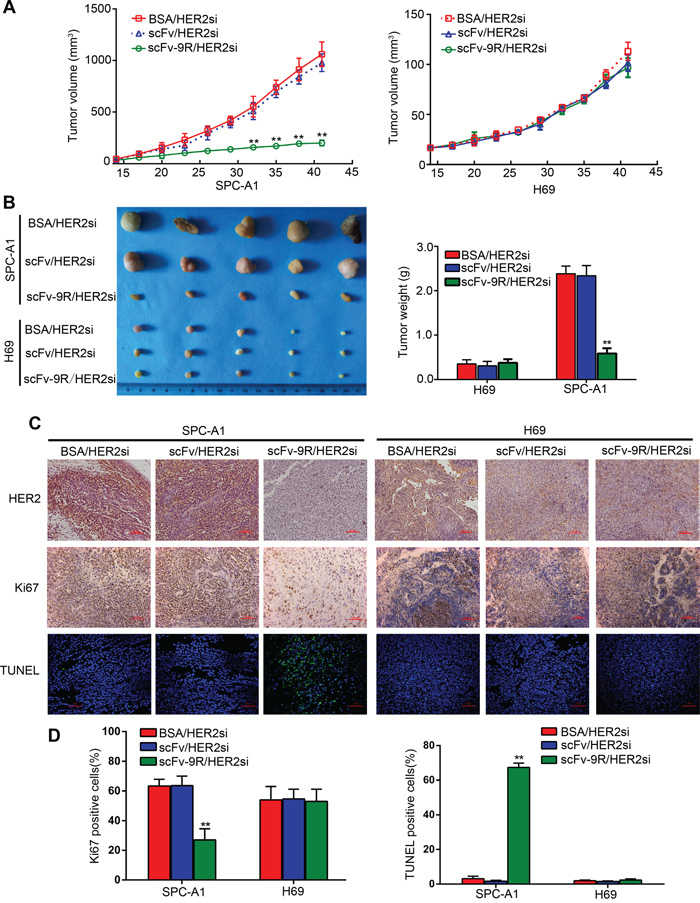
Intravenous injection of scFv-9R/HER2si complex suppressed xenograft tumor growth in nude mice Nude mice bearing SPC-A1 or H69 xenografts were intravenously treated with BSA/FAMsi, scFv/FAMsi or scFv-9R/FAMsi complex. **A.** Tumor volumes were calculated and presented as growth curves. **B.** Tumor nodules were collected, photographed and weighted after sacrificing mice at the end of the experiment. **C.** Tumor sections were stained for HER2, Ki67 and TUNEL as the representative microscopic images (×200) shown. Scale bar, 50 μm. **D.** Percentages of TUNEL or Ki67-positive cells from six randomly-selected fields of tumor paraffin-embedded sections were calculated. Data are expressed as mean ± S.D. for each group. **p < 0.01.

Finally, we evaluated the potential antitumor activity of scFv-9R/HER2si in EGFR-positive, HER2-overexpressed NSCLC using xenograft mouse model. Tumor bearing nude mice were treated with the BSA/HER2si, scFv/HER2si or scFv-9R/HER2si (2’-O-me modified) via intravenous injection biweekly up to six weeks. Thereafter, tumor growth was monitored and tumor tissue was examined. As shown in Figure 4A and 4B, treatment with scFv-9R/HER2si markedly suppressed growth of SPC-A1 xenografts in nude mice. But the tumor growth restraint was not observed in BSA/HER2si- and scFv/HER2si-treated SPC-A1 control groups. Moreover, scFv-9R/HER2si has no anti-tumor effect on EGFR-negative H69 cells, emphasizing the specificity of the anti-tumor effect of scFv-9R/HER2si on EGFR/HER2 dual positive tumors.

Immunohistochemistry (IHC) analysis exhibited reduced HER2 staining in tumor slices from SPC-A1 xenograft mice treated with scFv-9R/HER2si in comparison with the slices from scFv/HER2si- or BSA/HER2si-treated mice (Figure 4C). Moreover, the Ki-67 positive proliferating cell proportion was about 63.33 ± 4.51% and 63.53 ± 6.44% in BSA/HER2si and scFv/HER2si group, respectively; but the rate was much lower in scFv-9R/HER2si group (27.00 ± 7.55%) (Figure 4C and 4D). The percentage of TUNEL-positive cells was 67.33 ± 2.52% in tumor tissues from SPC-A1 xenograft mice treated with scFv-9R/HER2si. However, TUNEL-positive apoptotic cells were under 5% in either BSA/HER2si or scFv/HER2si group (Figure 4C and 4D). In addition, HER2, Ki-67 and TUNEL staining showed that scFv-9R/HER2si have no effect on H69 cells as for HER2 gene silencing, proliferation inhibition and apoptosis induction. Together, these data suggest that systematic administration of scFv-9R/HER2si significantly suppresses tumor growth by inhibiting cell proliferation and inducing cell apoptosis. Strikingly, when we stained the tumor slices with a monoclonal anti-CD31 antibody to evaluate the status of tumor angiogenesis, we found that tumors from SPC-A1 xenograft mice treated with scFv-9R/HER2si showed a lower level of CD31 expression than the ones from the control groups (Supplementary Figure 5). Moreover, *in vivo* toxicity was also evaluated as shown by tissue morphology (Supplementary Figure 6). ScFv-9R/HER2si was very well tolerated with no significant dose-dependent changes or effects on normal tissues.

### Expression and clinic-pathological correlation of HER2 in human NSCLC specimens

To confirm clinical relevance of HER2 expression in NSCLC, we used IHC to assess HER2 expression in NSCLC specimens obtained from bronchoscopic lung biopsy, and analyzed its correlation with clinic-pathological parameters. As shown in Table 1, among 75 patients included in this study, there were 21 HER2-positive cases accounting for 28% of total cases, including 11 adenocarcinoma, 8 squamous cell carcinoma, and 2 large cell lung cancer. The median survival time of all the patients was 16.3 months. Among them, 44 patients died in 1 year after diagnosis (58.7%) with 28 specimens being HER2 positive (63.6%); and 31 patients survived more than 1 year (41.3%) with only 3 cases being HER2 positive (9.7%). More importantly, the positive HER2 expression in NSCLC was significantly correlated with lymph node metastasis (*p*=0.000) and distant metastasis (*p*=0.001). Moreover, the median survival time for 18 patients (24%) with both EGFR and HER2 expression was only 6.3 months, suggesting that the patients with both genes expression may benefit from scFv-mediated HER2 silence strategy.

**Table 1:** Correlation between clinico-pathological parameters and HER2 expression

*Feature*	*N*	*HER2 positive N(%)*	*P value*
**Age (Year)**			0.719
<60	36	12(33.3)	
≥60	39	9(23.1)	
**Sex**			0.322
Male	51	8(15.7)	
Female	24	8(33.3)	
**Pathologic type**			0.074
Adenocarcinoma	37	11(29.7)	
Squamous cell carcinoma	32	8(25)	
Others	6	2(33.3)	
**Lymph node metastasis**			0.000
No	16	1(6.3)	
Yes	59	20(33.9)	
**Distant Metastasis**			0.001
No	30	2(6.6)	
Yes	45	19(45.2)	
**Survival time (months)**			0.000
<12	44	28(63.6)	
≥12	31	3(9.7)	

## DISCUSSION

In NSCLC, HER2 mutation, amplification or over-expression was regarded as a poor prognostic marker for survival, and conferred resistance to current EGFR-TKIs therapy [5, 6, 28]. The feature of HER2 is that its extracellular domain is in a constitutively active conformation even without ligand stimulation, which makes HER2 a preferred partner for dimerization with other HER family members and an attractive target for cancer treatment [29, 30]. In our analysis of 75 NSCLC cases, 28% of the tumor samples overexpress HER2 and there seems to be a trend that HER2 overexpression is more common in adenocarcinoma than in other histotypes (*p*=0.074), which awaits further confirmation in larger samples. Notably, high level of HER2 was significantly related to lymph node metastasis (*p*=0.000), distance metastasis (*p*=0.001), and shorter overall survival (*p*=0.000). These findings are consistent with previous studies showing that the expression of EGFR and HER2 protein in tumor tissue was more common in advance stage of NSCLC [9, 31], suggesting that targeting HER2 signaling may benefit NSCLC patients. However, clinical trials using HER2-directed agents as monotherapy in lung cancers showed disappointing results [32]. Moreover, although anti-HER2 treatment plus chemotherapy was shown to be associated with better clinical outcomes in patients with HER2 aberration, its application is limited because of severe side effects and frequent acquired resistance [33, 34]. Therefore, novel therapeutic approaches should be considered and included in the treatment of NSCLC.

As a promising agent for anti-cancer treatment, the efficacy of siRNA for gene silencing *in vivo* depends on whether it can successfully pass through cell membrane of the target cells [35]. Since most of the mammalian cells do not take up siRNA, developing an efficient delivery system is one of the major challenges in the field today [19]. Cellular uptake of siRNA via receptor-mediated endocytosis renders antibody-based delivery of siRNA, such as scFv/siRNA delivery system, as a potential anti-cancer therapeutics [36]. The size of scFv was much smaller than the intact antibody as they lack both the constant regions and the Fc domain, which means stronger penetrating capability, lower immunogenicity and toxicity [20]. Additionally, large scale manufacture of scFv could be accomplished with the engineered bacteria, so it is less time-consuming and expensive than production of full length antibody in mammalian cell expression systems. The ideal tumor antigen for scFv should be highly upregulated in tumor tissue, express on cell surface and be able to internalize into target cells [37]. Overexpressed EGFR is not only regarded as a driving event for NSCLC, but also a promising biomarker for antibody-based cancer treatments [38-40]. Nimotuzumab is an EGFR monoclonal antibody that has been approved as an orphan drug for the treatment of glioma and head and neck squamous cell carcinomas and in the process of clinical trials for other solid tumors including NSCLC [41, 42]. Nimotuzumab can trigger receptor-mediated internalization from cellular membrane to cytoplasm in EGFR-positive tumor cells [43]. Indeed, the recombinant scFv derived from Nimotuzumab retains EGFR-binding and internalization ability. In response to antibody binding, EGFR on cell surface will be phosphorylated and activated, subsequently resulting in receptor internalization and intracellular signaling [44]. The process of antibody-receptor complex internalization includes recruitment of adaptins and clathrin, inward budding of the plasma membrane, formation of early endosomes and finally trafficking to late endosomes and lysosomes [45]. Once inside lysosomes, scFv bound by the receptor will be degraded, and free siRNA carried by scFv will be released into the cytoplasm, leading to specific gene silencing.

With this powerful scFv-9R/siRNA delivery system, we successfully delivered HER2-siRNA into EGFR-positive NSCLC cells. As a result, HER2 gene expression was downregulated and cell growth were inhibited due to G1 arrest, which is consistent with our previous finding [46]. Because HER2 has the strongest catalytic kinase activity among all the four HER family members, HER2-containing heterodimers have the highest mitogenic potential among all HER complexes [47]. These heterodimers strongly recruit survival and mitogenic pathways, such as the mitogen-activated protein kinases (MAPK) and the phosphatidylinositol 3-kinase (PI3K) pathways, leading to dysregulation of the cell cycle homeostatic machinery [48, 49]. Our results show that scFv-9R/HER2si complex, through binding to EGFR homodimers or EGFR/HER2 heterodimers, internalize into EGFR-positive lung cancer cells and release HER2si, consequently leading to HER2 silence. HER2 knockdown attenuated downstream signaling of EGFR/HER2 heterodimers, thereby downregulating cell cycle-related gene expression and cell cycle-associated kinase activity and thus leading to cell accumulation at G1 phase [47, 50-52].

Using NSCLC cell-xenografted mice model, we further demonstrated that systematic injection of scFv-9R/HER2si resulted in a dramatic retardation of tumor growth. According to the histological analysis, scFv-9R/HER2si treatment not only led to cell proliferation inhibition, but also induced cell apoptosis. However, we did not observe obvious cell apoptosis *in vitro*, when the cells were treated with scFv-9R/HER2si (Supplementary Figure 2) or Lipofectamine/HER2si (data not shown). In the view of fact that HER2-targeted therapy can directly suppress angiogenesis by inhibiting vascular endothelial growth factor (VEGF) expression [53, 54], we speculated that scFv-9R/HER2si-mediated HER2 silencing might induce tumor cell apoptosis partially by hindering the process of vascularization in nude mice model, since HER2 expression status in xenografted tumors correlate to tumor microvessel density measured by CD31 immunohistochemistry. Importantly, scFv-9R/HER2si was very well tolerated and did not cause obvious treatment-related toxicity to animals.

In conclusion, our study provides preclinical support for the therapeutic potential of scFv-based RNAi therapy targeting HER2 in NSCLC management. More than twenty percent of NSCLC cases express HER2, indicating the therapeutic potential of the HER2-targeting strategy. ScFv-9R possesses highly EGFR-binding and internalizing activity, and is very effective in siRNA intracorporal delivery. scFv-9R/siRNA complex mediated HER2 knockdown exerted desirable anti-tumor effect both *in vitro* and *in vivo*. Therefore, this strategy might benefit a subset of NSCLC patients by site-directed eliminating HER2, the most potent member of the EGFR family from lung cancer cells.

## MATERIALS AND METHODS

### Cell lines

EGFR/HER2 dual-positive human lung adenocarcinoma cell lines SPC-A1 and PC9 were obtained from Shanghai Institutes for Biological Sciences (Chinese Academy of Sciences, Shanghai, China). EGFR-negative, human small cell lung cancer cell line H69 was supplied by Beijing Cell Resource Center (Chinese Academy of Medical Science, Beijing, China). The cell lines were cultured in RPMI-1640 supplemented with 10% fetal bovine serum (FBS, Gibco, Life Technologies, USA) at 37°C in 5% CO2.

### siRNA

HER2-siRNA (HER2si) and EGFR-siRNA (EGFRsi) oligonucleotide sequences are provided in Supplementary Table 1. All siRNAs used for *in vivo* experiments were modified with 2-O-methylation at all U and C residues of the sense strand to enhance stability, and reduce off-target and immunostimulatory effects. FAM-siRNA (FAMsi) was synthesized by modifying the 5′-end of the sense strand with fluorescein (GenePharma, Shanghai, China). Control groups were transfected with siRNA using Lipofectamine^2000^ (Invitrogen, Life Technologies, USA) according to the manufacturer's instructions.

### Expression and purification of EGFR-specific scFv and scFv-9R fusion protein (scFv-9R)

The recombinant plasmid pGEX-4T-1-scFv-9R, contains cDNA of an anti-EGFR scFv, and an nona-arginine residues (9R) linked to its C-terminus. scFv was designed according to the amino acid sequences of nimotuzumab (a monoclonal antibody against EGFR) with a linker (Gly4Ser3) connecting its VH and VL chains. pGEX-4T-1-S only contains cDNA of scFv fragment and served as a control. scFv-9R and scFv were fused with a 6×His.tag at N-terminus. Single colonies of *E. coli* BL21 (DE3) carrying the recombinant plasmid pGEX-4T-1-scFv-9R and pGEX-4T-1-S were grown overnight at 37°C in 2×YT medium supplemented with 100 μg/ml ampicillin. The cultures were diluted 100-fold in the same medium, and induced with 0.1 mM isopropyl-1-thio-β-galactopyranoside (IPTG, Wilson, China) at 30°C for 5 h. The fusion proteins were purified from the *E. coli* cell lysates with a glutathione-sepharose affinity column (GE Healthcare, Uppsala, Sweden) after 30 min of sonication. The GST tag was cleaved by the 25-fold diluted thrombin (Novagen, Darmstadt, Germany) at room temperature for 8 h, and removed from the solution by GST affinity chromatography.

### Affinity for cellular EGFR and targeted delivery of FAM-labeled siRNA (FAMsi) assessed by FCM

SPC-A1, PC9 and H69 cells were incubated in medium containing 10 μg scFv or scFv-9R at 37°C for 6 h, followed by an incubation with the anti-His mouse monoclonal antibody (#34660, Qiagen, CA, USA) at room temperature for 2 h. The cells were then stained with FITC-labeled goat anti-mouse secondary antibody (Boster, Wuhan, China) at 4°C for 1 h. After washing with ice-cold PBS, the fluorescence intensity of cells was recorded using a FACSCalibur flow cytometer (BD Bioscience, Mountain View, CA, USA). Data from 10,000 cells were collected and analyzed using the Cell-Quest software (BD Bioscience). To examine the targeted delivery of siRNA, 10 μg scFv, scFv-9R or BSA were pre-mixed with 20 nM FAMsi at room temperature for 30 min and then added into the culture medium of SPC-A1, PC9 and H69 cells. Six hours later, the cells were harvested and washed with cold PBS extensively and then analyzed by FCM. Transfection of FAMsi using Lipofectamine served as a positive control.

### Translocation of single chain antibodies and siRNA analyzed by laser scanning confocal microscopy

SPC-A1, PC9 and H69 cells were plated onto glass cover-slips and cultured at 37°C overnight. H69 cells were adhered to the cover-slips by centrifugation at 1000×g for 5 min. Cells were then incubated with 10 μg of either scFv or scFv-9R at 37°C for 6 h. After being fixed in 4% (W/V) paraformaldehyde in PBS for 15 min, the samples were incubated with anti-His mouse monoclonal antibody at room temperature for 2 h, washed extensively, and then incubated with Cy3-conjugated goat anti-mouse secondary antibody (Boster, Wuhan, China) for 1 h. The cell nuclei were counterstained with 4, 6-diamidino-2-phenylindole dihydrochloride (DAPI, Sigma-Aldrich, St. Louis, USA) in a 1.5 μg/ml solution at room temperature for 5 min. After extensive wash, cellular uptake and intracellular distribution of scFv and scFv-9R were analyzed by red fluorescence with laser scanning confocal microscopy (LSCM, A1, Nikon, Japan). To examine scFv/scFv-9R-mediated delivery of siRNA, 20 nM FAMsi were pre-mixed with scFv, scFv-9R or BSA at room temperature for 30 min, and then added to the cell culture medium. Six hours later, green fluorescence of FAMsi within the cells were visualized and imaged under LSCM. FAMsi transfected using Lipofectamine served as a positive control.

### Reverse transcription and quantitative real-time polymerase chain reaction (qRT-PCR)

Total RNA was isolated using a TRIzol reagent kit (Invitrogen, CA, USA), and cDNA was synthesized using 1 μg RNA with the PrimeScript™ RT Master Mix kit (Takara, Dalian, China) according to the manufacturer's instructions. The cDNA was used as a template for PCR. We designed specific primers by using the Primer Express 1.0 software (Applied Biosystems, CA, USA) based on human HER2 and β-actin mRNA sequences from Genbank: HER2 (forward) 5′-TGGCCTGTGCCCACTATAAG-3′ and (reverse) 5′-AGGAGAGGTCAGGTTTCACAC-3′; β-actin (forward) 5′-AGCGAGCATCCCCCAAAGTT-3′ and (reverse) 5′-GGGCACGAAGGCTCATCATT-3′. qRT-PCR was performed using a CFX96™ Real-Time PCR system (BioRad, Valencia, USA) with SYBR Green reagents (^#^DRR041A; Takara, Dalian, China) according to the manufacturer's instructions. Details are provided in the Supplementary methods.

### Western blot

For assessing HER2 expression and EGFR pathway signaling, lung cancer cells were lysed directly using RIPA buffer (150 mM NaCl, 1% NP40, 0.1% SDS, 1 mM PMSF, 0.5% sodium deoxycholate and 1 mM sodium orthovanadata). The proteins in cell lysates were quantified by bicinchoninic acid assay (Pierce, Rockford, IL, USA). Forty micrograms of total protein were separated on an 8% SDS–PAGE gel. Proteins were then transferred onto nitrocellulose membranes (Millipore, Bedford, USA) and incubated with the rabbit anti-HER2/ErbB2 polyclonal antibody (1:1000, #18299-1-AP, Proteintech, Wuhan, China), rabbit polyclonal anti-ERK1/2 antibody (1:1000, #11257-1-AP, Proteintech, Wuhan, China), rabbit monoclonal anti-phospho-ERK1/2 antibody (1:1000, Thr202/Tyr204, #4370, Cell Signaling, MA, USA), rabbit polyclonal anti-Akt antibody (1:1000, #10176-2-AP, Proteintech, Wuhan, China) and rabbit monoclonal anti-phospho-Akt antibody (1:1000, Ser473, #ab81283, Abcam, Cambridge, UK), followed by the horseradish peroxidase (HRP)-conjugated goat anti-mouse IgG secondary antibody or goat anti-rabbit IgG secondary antibody (1:10000, Genshare, Xi’an, China). Finally, ECL detection reagent (Genshare, Xi’an, China) was used to visualize the protein bands.

### Assessment of cell cycle distribution by FCM analysis

1 × 10^6^ of lung cancer cells were harvested and re-suspended in 500 μL of PBS. After being fixed with 500 μL cold 100% ethanol at 4°C overnight, cells were stained with 200 μL PI (50 μg/mL, Sigma-Aldrich, St. Louis, USA) at room temperature for 20-30 min. Cell cycle distribution was determined by fluorescence-activated cell sorting analysis with FCM. Each assay was performed in triplicate and the experiment was repeated at least three times independently.

### Cell viability assay and colony-formation assay

Cells in logarithmic growth were seeded in 96-well plates (3 × 10^3^ cells per well) in 200 μL medium supplemented with 10% FBS and incubated in a 37°C humidified and 5% CO2 incubator for 1-10 days. During this period, the cells were repeatedly treated with the fusion protein/HER2si complexes (or control) every 48 hours. The cell viability was assessed by MTT assay every day. Briefly, 20 μL of 5 mg/ml MTT (Sigma-Aldrich, St. Louis, USA) was added to each well, and cells were incubated for another 4 h followed by the addition of 150 μL DMSO (Sigma-Aldrich, St. Louis, USA) for 20 min. The absorbance values at 490 nm were determined using the microplate reader.

For colony-formation assay, the cells were seeded in 6-well plates (200-500 cells/well) in 2 mL medium with 10% FBS, cultured and treated with the fusion protein/HER2si complexes as above for up to 12 days. The resultant colonies were fixed with 100% methanol for 15 min, and then stained them with Giemsa (Sigma-Aldrich, St. Louis, USA) at room temperature for 15 min. The number of colonies larger than 50 μm in diameter were photographed and counted. Each assay was performed in triplicate and the experiment was repeated on at least three times independently.

### *In vivo* delivery of siRNA

All animal experiments were approved by the Animal Experiment Administration Commission of FMMU. The lung cancer xenograft model was established by injecting 5 × 10^6^ SPC-A1 or 10^7^ H69 cells/mouse into the thigh of BALB/c nude mice (4-6 weeks old, male, body weight 20-30 g). FAMsi (modified by 2’-O-methylation) were pre-incubated with scFv, scFv-9R or BSA at room temperature for 30 min, and then injected into the tumor-bearing mice via the tail vein. For each mouse, 40 μg of FAMsi was administered to at a molar ratio of fusion protein to siRNA as 1:3. Forty-eight hours after the injection, we isolated tumor specimens and normal tissues, prepared frozen sections and visualized FAMsi signal with LSCM.

### *In vivo* anti-tumor activity

BALB/c nude mice were injected with SPC-A1 and H69 cells as described above. Complexes containing 40 μg of HER2si and 1 nmol of scFv-9R, scFv or BSA in 200 μL of PBS, which were pre-mixed as described above, were injected into nude mice via the tail vein biweekly. Tumor volume (TV) were calculated twice a week for 6 weeks according the formula TV (mm^3^) = length×width^2^×0.5. After that, tumor tissues were harvested, weighed and formalin-fixed for paraffin sectioning. The tumor-tissue sections were stained using rabbit anti-HER2/ErbB2 polyclonal antibody, rabbit anti-Ki-67 monoclonal antibody (1:100, #BM2889, Boster, WuHan, China) or rabbit anti-CD31 polyclonal antibody (1:50, #ab28364, Abcam, Cambridge, UK), and the immunostained images were analyzed with a microscope (DM4000, Leica, German). To detect apoptosis in situ, paraffin-embedded sections of tumor tissues were labeled using a fluorescein-TUNEL kit (Roche, Lewes, UK) according to the manufacturer's instructions. Cells were identified by their centrally located nuclei, which were counterstained with DAPI. Labeled nuclei were counted to determine the apoptotic index.

### Clinical relevance of HER2 expression in NSCLC patients

A total of 75 human paraffin-embedded NSCLC tissue sections storage at the Department of Pathology in Xijing Hospital, were investigated for HER2 expression by immunohistochemistry. The association between HER2 expression and clinico-pathological parameters was examined by χ2 test. Details were provided in Supplementary methods.

### Statistical analysis

All experiments were performed in triplicate on three independent occasions and data are expressed as mean ± S.D. One-way ANOVA were employed for statistical analysis by SPSS 15.0 (SPSS, Chicago, USA). The differences between means were tested by an independent sample *t*-test. A *p* value < 0.05 was considered significant.

## SUPPLEMENTARY FIGURES AND TABLES


