# Supplementary material for: HER2-siRNA delivered by EGFR-specific single chain antibody inhibits NSCLC cell proliferation and tumor growth
Source: Oncotarget. 2016 Mar 14;7(17):23594–607. doi: 10.18632/oncotarget.8053 (PMC5029650; doi:10.18632/oncotarget.8053)
Supplement: Supplementary file 1 [file oncotarget-07-23594-s001.pdf]

HER2-siRNA delivered by EGFR-specific single chain antibody inhibits NSCLC cell proliferation and tumor growth

SUPPLEMENTARY DATA

SUPPLEMENTARY METHODS

Plasmid construction and protein purification

The genes encoding scFv and scFv-9R were designed according to the amino acid sequences of Nimotuzumab with a linker (Gly4Ser3) connected its VH and VL chains, and the nine-mer arginine encoding sequence designed in the 3′ scFv sequence to obtain the scFv-9R fusion gene. In order to label the scFvs, we also designed an 6×His.tag-encoding sequence at the 3′ scFv sequences, which was synthesized by Augct Co. (Beijing, China). For bacterial expression, the scFv and scFv-9R sequence were isolated as a BamHI/XhoI fragment from T vector and inserted the sequence into a BamHI/XhoI-digested plasmid, pGEX-4T-1 (Novagen, Uppsala, Sweden). Single colonies of *E. coli* BL21 (DE3) carrying the manufactured plasmid pGEX-4T-1-scFv for the expression of EGF receptor-specific scFv protein, were grown overnight at 37°C in 2×YT medium supplemented with 100 μg/ml ampicillin. We diluted the cultures 100-fold in the same medium, and incubated them at 37°C to an OD600 of 0.4-0.6, followed by inducing with 0.1 mM isopropyl-1-thio-β-galactopyranoside (IPTG) at 30°C for 5 h. Via centrifugation (5,000 g, 4°C, 15 min), cells were harvested, washed in ice-cold phosphate buffer saline (PBS), then re-suspended them in binding buffer (140 mM NaCl, 2.7 mM KCl, 10 mM Na2HPO4, 1.8 mM KH2PO4, pH 7.4). Soluble cell lysates was centrifuged at 12,000 g for 20 min after 30 min of sonication. Following the manufacturer's instructions, we then applied a glutathione-sepharose affinity column (GE Healthcare, Uppsala, Sweden) to the supernatant. The fusion proteins was eluted from the GST-affinity column using 10 mM reduced glutathione dissolved in 50 mM Tris-HCl (pH 8.0) at room temperature. We performed dialysis on the purified sample against PBS (PH 8.0) for 24 h at 4°C, then incubated the sample with 25-fold diluted thrombin (Novagen, Darmstadt, Germany) at room temperature for 8 h. Finally, the solutions were filtered in a glutathione-sepharose affinity column to remove the GST.tag. The resultant fusion proteins were quantified by a bicinchoninic acid assay (Pierce, Rockford, IL USA) and immuno-blotting with an anti-6×His.tag mouse monoclonal antibody (1:1000, #34660, Qiagen, CA, USA).

The actual sequence of the scFv peptide is as follows:

LQQSGAEVKKPGSSVKVSCKASGYTFTNYYI
YWVRQAPGQGLEWIGGINPTSGGSNFNEKFKTRVT
ITADESSTTAYMELSSLRSEDTAFYFCTRQGLWFDS
DGRGFDFWGQGTTVTVSGGGGSGGGGSGGGGSIQ
MTQSPSSLSASVGDRVTITCRSSQNIVHSNGNTYLD
WYQQTPGKAPKLLIYKVSNRFSGVPSRFSGSGSGT
DFTFTISSLQPEDIATYYCFQYSHVPWTFGQGTKLQI

Gel retardation assay

Ten nanogram random DNA fragments prepared by PCR using 2×Taq MasterMix (Cwbio, Beijing, China) were incubated with increasing amounts of the scFv (1μg), BSA (1μg), or scFv-9R (1, 3 and 7μg) at room temperature for 30 min before being run on an 0.8% agarose gel.

Affinity of the fusion proteins to EGFR measured by ELISA

The affinity of the scFv and scFv-9R fusion protein for EGFR was measured by enzyme-linked immunosorbent assay (ELISA). Briefly, recombinant EGFR (Proteintech, Wuhan, China; 1 μg per well) was immobilized on 96-well microplates and incubated with 100 nM of each the fusion proteins or BSA at 37°C for 1 h. Successively immunobloted with anti-6×His.tag antibody for 2 h and HRP-conjugated Goat Anti-Rabbit IgG (Boster, Wuhan, China) for 1 h. After extensive washing, a 3, 3′, 5, 5′-Tetramethylbenzidine (TMD) substrate was added and reaction was terminated after 30 min by adding 2 M sulfuric acid. The plates were read in the microplate reader at the absorbance of 450 nm. (Bio-Rad iMark680, California, America).

Reverse transcription quantitative real-time polymerase chain reaction (qRT-PCR)

The qRT-PCR analysis was performed in a total volume of 20 μL with the following amplification steps: an initial denaturation step at 95°C for 3 min, followed by 54 cycles of denaturation at 95°C for 10 s, anneal at

56°C for 15 s and extention at 72°C for 15 s. Data were analyzed according to the comparative Ct method and were normalized by β-actin expression in each sample. Data shown are representative of the mean values and standard deviations from three independent experiments performed in triplicate.

Expression and clinico-pathological correlation of HER2 in human NSCLC specimens

A total of 75 human paraffin-embedded NSCLC tissue sections storage at the Department of Pathology in Xijing Hospital, were investigated for HER2 expression by immunohistochemistry. The deparaffinized specimens were incubated with rabbit anti-human HER2 monoclonal antibody (1:100, #BA0015-1, Boster, Wuhan, China) overnight at 4°C and detected by the IHC rabbit DAB kit (NeoBioscience, Beijing, China).

HER2-positive NSCLC specimens were further selected for clinico-pathological correlation analysis. Clinical parameters such as gender, age, lymph node metastasis and TNM stage were collected. The association between HER2 expression and clinico-pathological parameters was examined by χ^2 test. A p value of <0.05 was considered significant. Patients offering samples for the study signed informed consent forms. This study was approved by the Moral and Ethical Committee of Fourth Military Medical University.

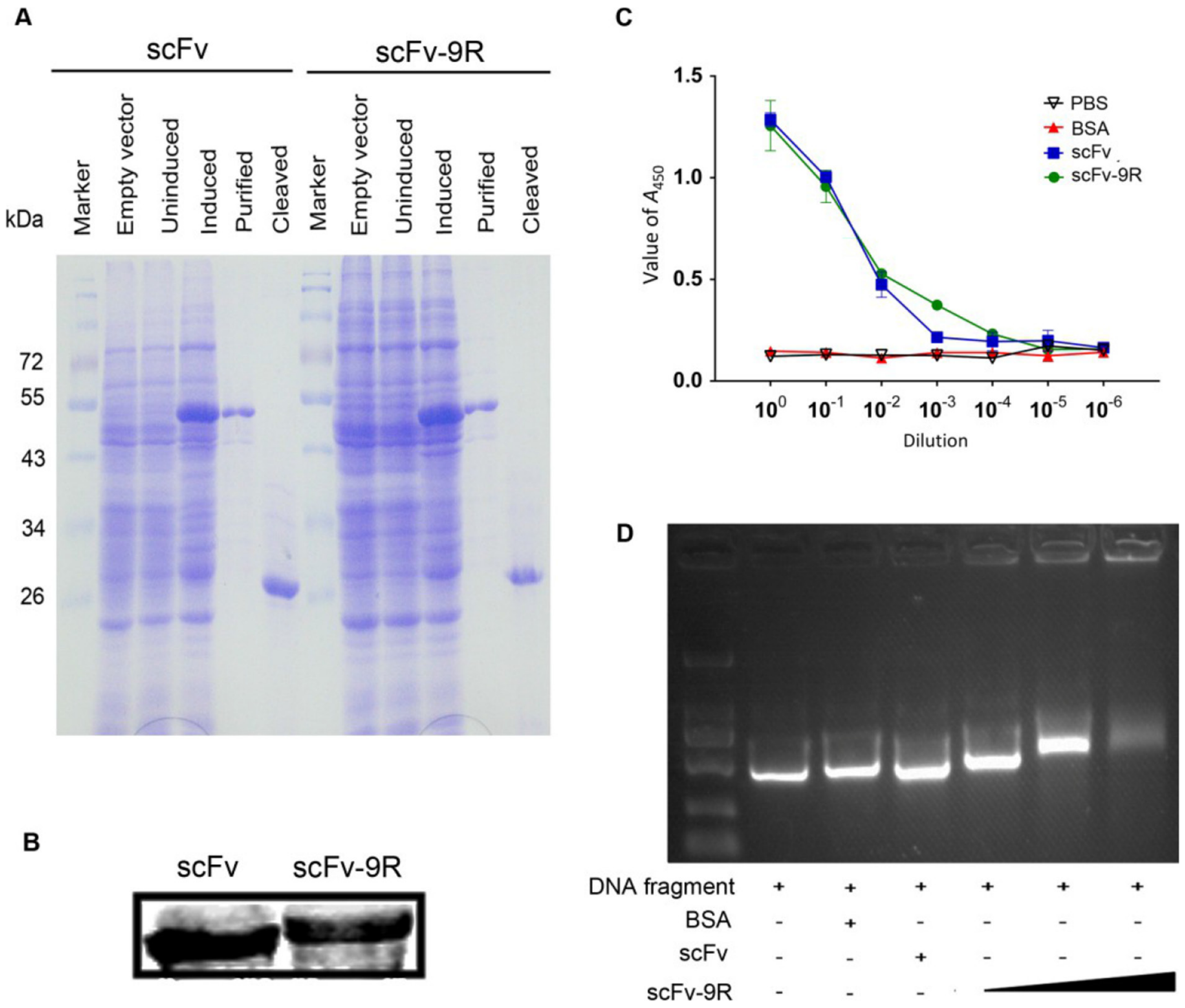

Supplementary Figure 1: Expression and purification of scFv-9R and scFv. A. Expressed and purified of scFv-9R and scFv fusion proteins. **B.** Identification of the purified scFv-9R and scFv fusion proteins by westernblot. **C.** The EGFR-binding ability of scFv-9R and scFv detected by ELISA. **D.** Nucleotide binding ability of the scFv-9R and scFv fusion proteins determined by gel mobility-shift assay.

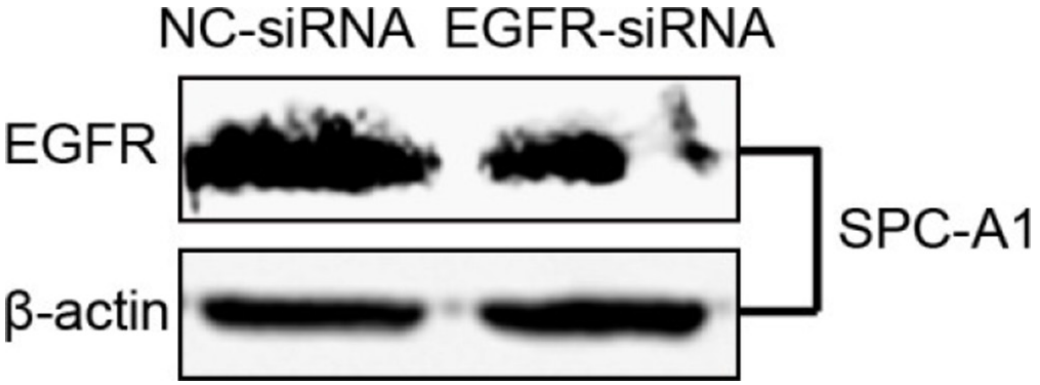

Supplementary Figure 2: Silence efficiency of EGFR-siRNA was test by Western blot in SPC-A1 cells.

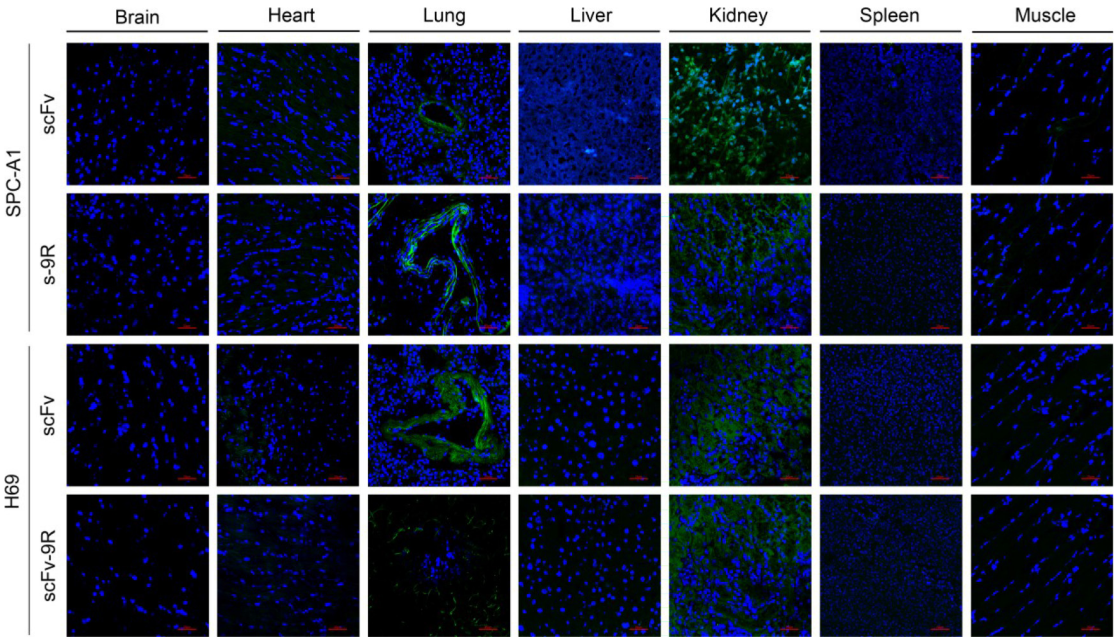

Supplementary Figure 3: The localization of FAMsi in different tissues observed by LSCM in freshly frozon tissue sections. Scale bars, 20 μm.

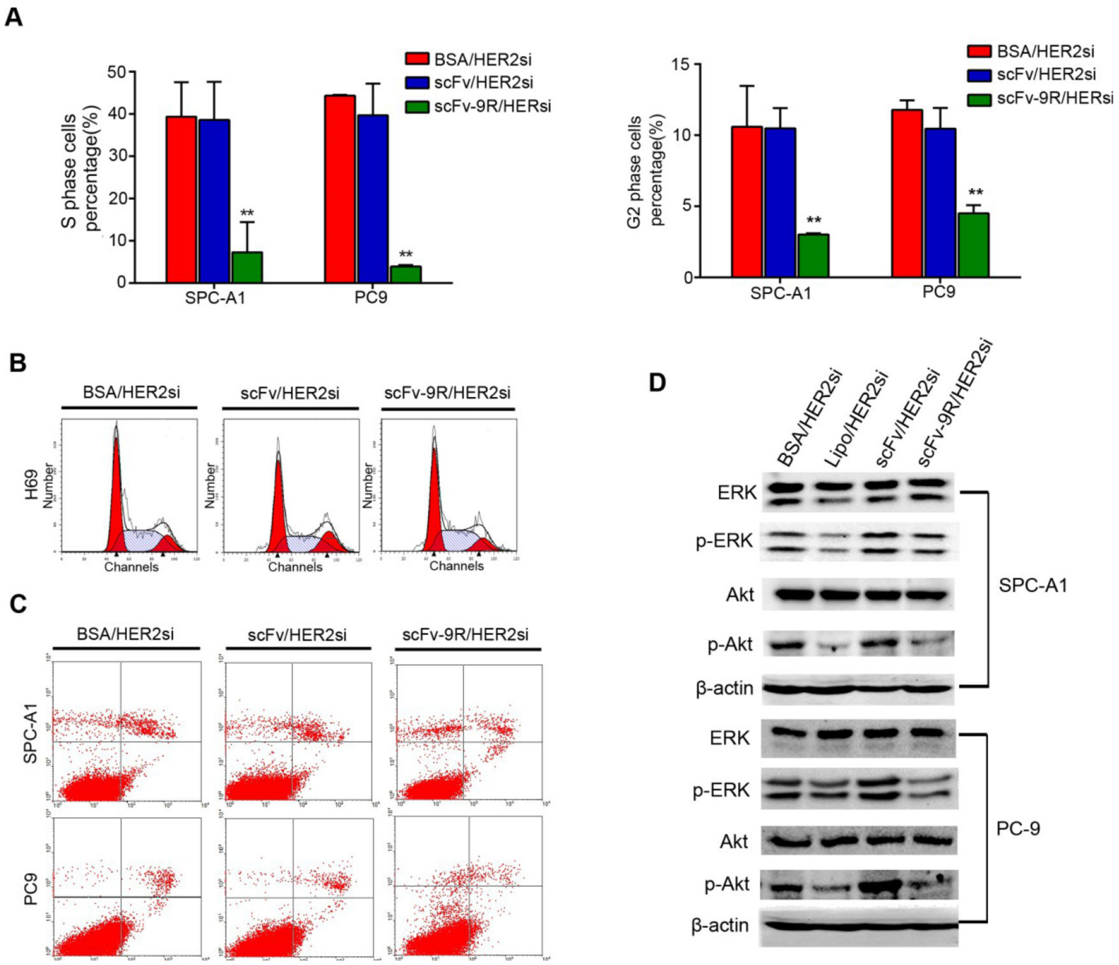

Supplementary Figure 4: A. FCM analysis of cell cycle distribution of SPC-A1 and PC9 cells treated as indicated. **B.** FCM analysis of cell cycle distribution of H69 cells treated as indicated. **C.** FCM analysis of cell apoptosis of SPC-A1 and PC9 cells treated as indicated. **D.** effects of scFv-9R/HER2si on the phosphorylation status of HER2 downstream signaling.

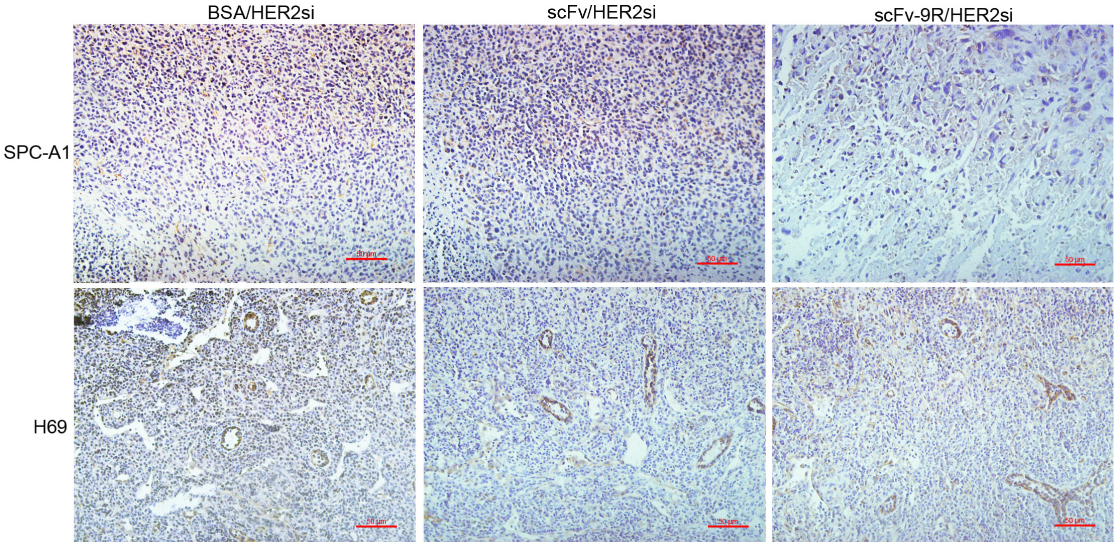

Supplementary Figure 5: Tumor sections were stained for CD31 with the representative microscopic images (×200) shown. Scale bar, 50 μm.

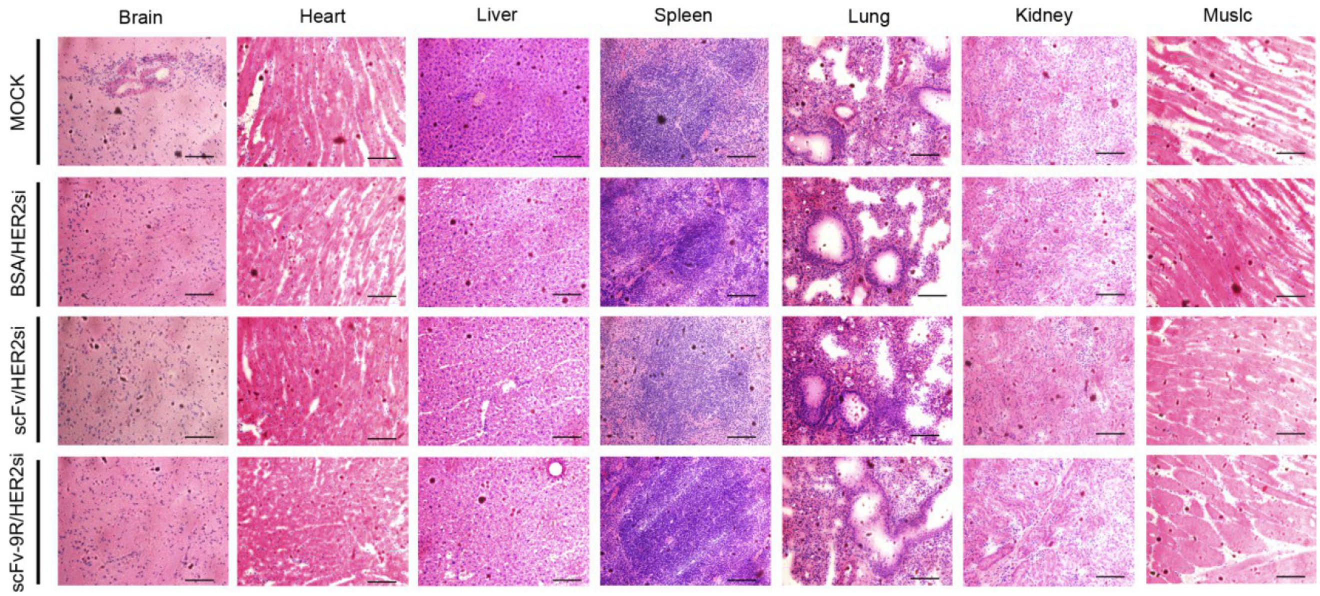

Supplementary Figure 6: Undetectable toxicity of repeated dosing of scFv-9R/HER2si. Morphological analysis of primary tissues was done by H&E staining (×200). Scale bar, 50 μm.

Supplementary Table 1: Sequences of siRNAs

siRNA	Sequences (5′-3’)
HER2-siRNA	Sense 5’-UGAAACCUGACCUCUCCUATT-3’
	Antisense 5’-UAGGAGAHHUCAGGUUUCATT-3’
EGFR-siRNA	Sense 5’-CGUCGCUAUCAAGGAAUUATT-3’
	Antisense 5’-UAAUUCCUUGAUAGCGACGTT-3’
NC-siRNA	Sense 5’-UUCUCCGAACGUGUCACGUTT-3’
	Antisense 5’-ACGUGACACGUUCGGAGAATT-3’
